# The Sound of Memory: Investigating Music Therapy’s Cognitive Benefits in Patients with Dementia—A Network Meta-Analysis of Randomized Controlled Trials

**DOI:** 10.3390/jpm14050497

**Published:** 2024-05-08

**Authors:** Berne Ting, Chen-Hsin Su, Daniel Tzu-Li Chen, Wei-Ti Hsu, Chia-Lin Tsai, Pan-Yen Lin, Li Jingling

**Affiliations:** 1 Ph.D. Program for Aging, College of Medicine, China Medical University, Taichung 404328, Taiwan; u109048801@cmu.edu.tw; 2 Department of Psychiatry, Chiayi Christian Hospital, Chia-Yi 600566, Taiwan; u9701052@cmu.edu.tw; 3 M.D.-Ph.D. Program, College of Medicine, China Medical University, Taichung 404328, Taiwan; u105023415@cmu.edu.tw; 4 Graduate Institute of Biomedical Sciences, College of Medicine, China Medical University, Taichung 404328, Taiwan; u108305203@cmu.edu.tw (W.-T.H.); u9702602@cmu.edu.tw (C.-L.T.); 5 Mind-Body Interface Laboratory (MBI-Lab), China Medical University Hospital, Taichung 404327, Taiwan; 6 Department of Anesthesiology, China Medical University Hospital, Taichung 404327, Taiwan; 7 Department of Psychiatry, Wei Gong Memorial Hospital, Miaoli 351498, Taiwan

**Keywords:** dementia, cognitive, randomized controlled trials, music therapy, music medicine, music psychology, music intervention

## Abstract

Numerous previous studies have shown the effectiveness of music therapy in enhancing cognitive functions in patients with dementia. Despite this, robust evidence in this field, especially concerning the comparison of different music therapy types, is lacking. Therefore, randomized controlled trials (RCTs) focusing on music therapy and cognitive functions in dementia patients, termed by “music” AND “dementia” OR “Alzheimer’s disease” AND “cognitive”, were identified from primary electronic databases to conduct this network meta-analysis (NMA). The primary outcome focused on the impact on cognitive functions, and the secondary outcome was the comparison of dropout rates between the intervention groups and the usual care control groups. Standardized mean difference (SMD) values and the corresponding 95% confidence intervals (CIs) were computed for effect evaluation. This study protocol has been registered in IPLASY (INPLASY202430082). A total of 14 RCTs with 1056 participants were enrolled, examining interventions including Active Music Therapy (AMT), Active Music Therapy with Singing (AMT + Sing), Rhythmic Music Therapy (RMT), Listening to Music (LtM), and Singing (Sing). The results indicated that RMT, AMT + Sing, and AMT all significantly improve cognitive functions in dementia patients, of which the SMD were 0.76 (95% CI = 0.32–1.21), 0.79 (95% CI = 0.03–1.49), and 0.57 (0.18–0.96), respectively. Compared with the control group (usual care), no music therapy type was associated with an increased dropout risk. In conclusion, music therapy can improve cognitive functions in patients with dementia without increasing the risk of dropout, particularly RMT, AMT + Sing, and AMT.

## 1. Introduction

Dementia is an increasingly prevalent neurodegenerative disorder worldwide, characterized by cognitive, behavioral, and functional impairments in patients [1]. Cognitive function refers to the brain’s capacity to carry out various mental activities, including learning, thinking, memory, language, problem-solving, decision-making, and attention. These abilities are crucial for self-care, communication, work, and social interaction in daily life [2]. By contrast, the impartment of cognitive function may be a critical problem for dementia patients’ quality of life (QoL). However, treating this condition poses a significant challenge due to the involvement of various brain regions and extensive neural networks. This complexity can result in damage or functional decline in multiple bodily systems, which substantially complicates the diagnostic [3]. Current dementia treatment strategies aim to alleviate symptoms and enhance QoL and functional capabilities. These include pharmacological therapies, such as cholinesterase inhibitors and N-Methyl-D-Aspartate receptor antagonists, which may also benefit patients experiencing behavioral symptoms [4,5,6]. Additionally, non-pharmacological approaches are employed, including cognitive therapy, behavior management, psychosocial interventions, daily living skills training, and creative therapies such as art and music [7,8].

Music therapy has been shown to enhance psychological, physical, and emotional well-being, improve mood [9], reduce pain and anxiety [10,11], and elevate the overall QoL in patients with dementia. This therapy involves diverse activities such as listening, playing, composing, and improvising music, aiming to foster self-expression, social interaction, and motor skills [12]. It leverages rhythm and melody to affect brain waves, heart rate, and respiration, benefiting emotional and physiological states [13,14]. Additionally, music therapy promotes neuroplasticity, enhancing brain connectivity, which is particularly beneficial for activating memory-related areas like the hippocampus in dementia patients, thereby improving cognitive functions and emotional conditions [15]. Despite these benefits, the optimal types of music therapy for significant outcomes remain unclear. Therefore, identifying the most effective music therapy approaches is crucial for developing targeted treatment plans for dementia patients [16,17].

Network Meta-Analysis (NMA) represents a statistical methodology designed to evaluate multiple interventions concurrently, enabling the determination of the most efficacious therapies [18]. As the highest level of evidence, NMA plays an important role in clinical strategy and practice. This technique initially entails the compilation and systematic organization of diverse established interventions in music therapy. Subsequently, a network model is constructed for the comparative evaluation of these interventions, ordering them based on their effectiveness. Direct comparisons occur in studies that explicitly contrast various interventions. Where direct comparisons are absent, indirect comparisons are drawn using a shared comparator. NMA rigorously examines statistical differences in comparative assessments that encompass direct and indirect evidence, ensuring coherence and reliability within the analysis [19,20]. This study aims to establish a clear ranking of music therapy interventions based on their effectiveness in enhancing cognitive function in dementia. Identifying and selecting the most impactful music therapy interventions is crucial, ensuring that therapeutic strategies are both targeted and effective, thereby improving the outcomes of dementia care.

## 2. Methods and Materials

This research was conducted meticulously adhering to the guidelines of the Preferred Reporting Items for Systematic Reviews and Meta-Analyses extensions, specifically for Network Meta-Analysis (PRISMA NMA) [21]. The study protocol was properly registered with the International Platform of Registered Systematic Review and Meta-analysis Protocols (INPLASY), registration ID: INPLASY202430082.

### 2.1. Database Searches and Study Identification

An exhaustive search across four electronic databases—PubMed, Web of Science, Embase, Cochrane Library and the Web of Science—was executed to identify pertinent studies. This search covered a period through January 2024. We employed Boolean operators with the terms: “music” AND “dementia” OR “Alzheimer’s disease” AND “cognitive”. This approach was designed to review and synthesize research on the effects of music therapy interventions on cognitive function in dementia. The initial phase involved screening to eliminate duplicates and exclude studies that were not primarily focused on cognitive function in dementia. Subsequently, a search was conducted manually, and the reference lists of several review articles [22,23,24,25,26,27,28] were examined for additional relevant studies. The titles and abstracts of the screened articles were then evaluated for relevance by two independent reviewers (Ting and Su). When disagreements arose between the reviewers, a third party (Li) intervened to facilitate consensus and finalize the selection process. This systematic approach ensured that every study incorporated into the review was relevant and satisfied the predefined eligibility criteria.

### 2.2. Inclusion and Exclusion Criteria

This NMA utilized the PICO framework (Population, Intervention, Comparison, and Outcome): P—individuals with dementia; I—music therapy; C—control group without intervention; and O—established measures for assessing cognitive function in dementia. Studies fitting these criteria were included: (1) Studies were performed as randomized controlled trials (RCTs); (2) Intervention groups were treated with music therapy and music intervention encompassing rhythm, melody, and harmony, whereas control groups were given standard care, absence of treatment, or non-music intervention; (3) Outcomes were measured using cognitive function assessment scales; and (4) Participants in the study were diagnosed with dementia, mild cognitive impairment (MCI) or had self-reported memory loss. Exclusion criteria were as follows: (1) various types of publications including medical protocols, conference papers, review articles, pilot studies, preliminary findings from current research, case reports, editorials, and letters; (2) studies in which music therapy was combined with alongside other therapies, or regarded as a complementary or alternative therapy; (3) control groups involving any form of music; and (4) studies lacking a primary outcome analysis. The final selection for the NMA was based on the complete texts of the eligible articles.

### 2.3. Network Meta-Analysis Model Development

In our NMA, we meticulously structured the model based on specific guidelines. To reduce heterogeneity, we focused on pairwise comparisons exclusively between music therapy and other music therapy forms, or music therapy and standard care. We deliberately excluded comparisons involving music therapy with more invasive treatments, such as electrotherapy or laser light injections, as well as with nutritional supplements. Broadening the scope to include these treatments could have created diverse geometries of the network due to the various types of interventions involved, potentially yielding unreliable outcomes in the NMA [29]. The classification of different music types was established through collaborative discussions between two authors (Ting and Su), focusing on the specific content of music prescriptions. In instances of disagreement regarding the categorization, a third author (Li) was consulted to facilitate a discussion and achieve a unified consensus.

### 2.4. Methodological Quality Assessment

The methodological quality of the studies included was evaluated using the Cochrane Collaboration’s Risk of Bias Tool for Randomized Trials (RoB 2, version 2, London, UK) [30]. This tool thoroughly evaluates essential aspects of research quality, including the randomization process, adherence to intervention protocols, management of missing outcome data, the precision of outcome measurement, the likelihood of selective reporting, and the overall risk of bias in the study.

### 2.5. Primary Outcome: Cognitive Improvement in Patients with Dementia

Our main result was the enhancement of cognitive abilities in dementia patients, evaluated through the standardized mean difference (SMD). Considering its proven importance in evaluating cognitive function, the Mini-Mental State Examination (MMSE) [31] was the preferred scale for measurement. Other scales, such as the Montreal Cognitive Assessment (MoCA) [32] and the Frontal Assessment Battery (FAB) [33], were considered as secondary options. Additional cognitive assessment tools pertinent to dementia were also included as alternative measures. This structured approach in selecting scales was implemented to ensure uniformity and accuracy in assessing cognitive function throughout the study population.

### 2.6. Secondary Outcome: Differential in Dropout Rates

The secondary objective of this study was to assess the risk difference in dropout rates between participants undergoing music therapy and those in the control group. The ‘risk difference’ refers to the absolute difference in the proportion of participants who dropped out of the study in each group, providing a direct measure of participant retention. For example, if an intervention using music therapy to enhance cognitive functions in dementia records a 12% dropout rate, in contrast to a 7% rate in a control group (potentially engaged in unstructured music activities), the calculated risk difference would be five percentage points. This metric is vital for evaluating participants’ engagement level with the music therapy intervention and its feasibility in the context of dementia care. Such an assessment helps understand music therapy interventions’ comparative appeal and tolerability, as reflected through participant retention rates [34].

### 2.7. Data Extraction, Processing, and Transformation

The data extraction process, encompassing participant demographics, study designs, music therapy intervention specifics, and study outcomes, was independently executed by two researchers (Ting and Su). In cases where necessary data were absent in published studies, efforts were made to obtain this information directly from the studies’ authors. We adhered to data management protocols as outlined in the Cochrane Handbook, supplemented by guidance from the existing medical research literature [19,35,36,37,38]. This meticulous approach ensured uniform and careful handling of data, contributing to the reliability and validity of our NMA findings.

### 2.8. Statistical Analysis

We used a random-effects model to account for the diversity of music therapy types [39]. Using the frequentist method, the analysis was performed with MetaInsight (version 5.1.2; Complex Reviews Support Unit is funded by the National Institute for Health Research (NIHR), London, UK). The netmeta package in R is integrated into an online NMA platform for statistical analysis [40]. Initially, forest plots and network diagrams were generated to depict the pairwise comparisons in the studies. Following this, forest plots were generated to summarize the standardized mean differences (SMD) in cognitive function improvement and variations in dropout rates among elderly dementia patients. These plots juxtaposed the impact of each music therapy type against the control groups [41]. Outcomes were expressed as point estimates alongside 95% confidence intervals (95% CI) [41]. The music therapies were then ranked by effectiveness, and the results from both direct and indirect comparisons were displayed in tables. We assessed inconsistencies in the data using specific statistical tests, setting a two-sided *p*-value of less than 0.05 as the criterion for statistical significance.

### 2.9. Sensitivity Analysis Approach

We then conducted two distinct sensitivity analyses to validate the reliability of our results. The first analysis consisted of sequentially removing each study to assess whether any single study disproportionately influenced the overall findings. This method involved progressively removing each study and subsequently assessing and identifying how these removals influenced the ultimate conclusions and the interventions’ comparative effectiveness. The second sensitivity analysis concentrated on exploring the correlation coefficient applied in pre- and post-assessments of cognitive function. Initially, our study adopted a correlation coefficient of 0.8, adhering to the guidelines suggested in the Cochrane Handbook [35].

We conducted a further sensitivity analysis to acknowledge the variation in correlation coefficients used by researchers, typically falling between 0.5 and 0.8 [42]. In this analysis, we recalculated the effect sizes for changes in cognitive function using a lower coefficient of 0.5 [42], enabling us to evaluate how this change in the coefficient influenced the direction and magnitude of the outcomes, the statistical significance of these results, and the comparative efficacy of the interventions.

### 2.10. Publication Bias

We assessed the potential publication bias following the guidelines provided in the Cochrane Handbook for Systematic Reviews of Interventions [19]. A funnel plot targeting the comparisons involving the control group was generated using Comprehensive Meta-Analysis software, version 4 (Biostat, Englewood, NJ, USA), targeting the comparisons involving the control group. Additionally, to ascertain the presence of significant publication bias, we utilized the Egger’s regression test.

## 3. Results

### 3.1. Identification of Research and Construction of Network Models

**Figure 1:**
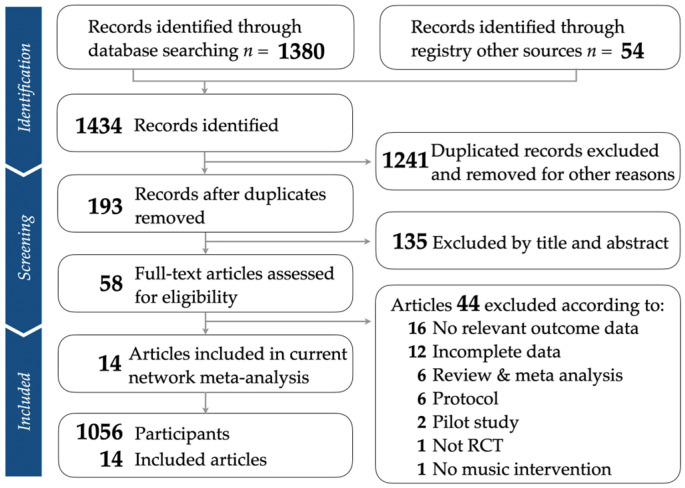
Flowchart depicting the process of the study selection, in accordance with PRISMA guidelines.

Our research rigorously adhered to the PRISMA guidelines, as illustrated in Figure 1. For additional information, the PRISMA NMA checklist can be found in Supplementary Table S1. The tally of articles sourced from different databases is detailed in Supplementary Table S2. Once duplicates were removed and studies not relevant based on titles and abstracts were excluded, we incorporated fourteen randomized controlled trials into our study [43,44,45,46,47,48,49,50,51,52,53,54,55,56]. Table S3 provides details on the articles excluded during the final selection phase, including the reasons for their exclusion.

**Figure 2:**
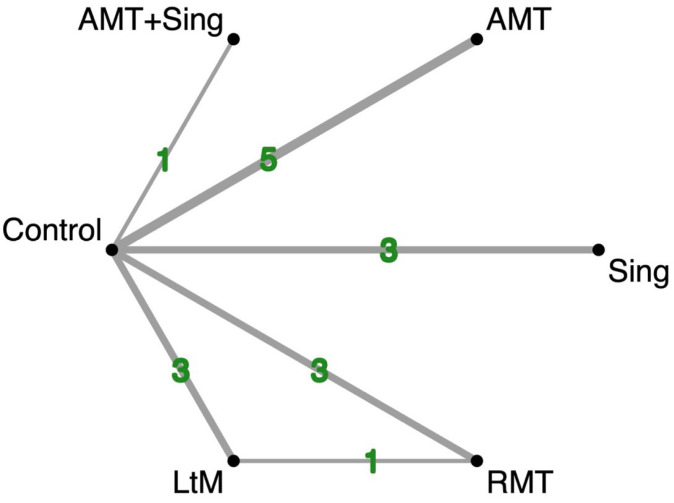
Network diagram showing the effects of various music interventions on enhancing cognitive function in dementia patients’ post-activity. The diagram’s node sizes and line thicknesses correspond to the number of trials included in our study. Abbreviations: AMT—Active Music Therapy; RMT—Rhythmic Music Therapy; LtM—Listening to Music; Sing—Singing.

In total, 14 randomized controlled trials were included, encompassing 1056 participants. The music interventions identified in these studies were classified into five categories: Active Music Therapy (AMT), Singing (Sing), Listening to Music (LtM), Rhythmic Music Therapy (RMT), and a combined method of AMT + Sing. A network model representing these various music therapy approaches is depicted in Figure 2.

This study’s general characteristics offer an extensive summary, including the authors, publication year, and originating country. The design of this study is elaborated upon, providing a clear understanding of the employed methodologies. Emphasis is placed on both the intervention and control groups, documenting key details like participant numbers, dropout rates, average age, dementia severity, and specific elements of the music therapy (such as session style, music types, and genres). Information regarding the control group, including the nature and descriptions of control strategies, is also included. Furthermore, this study examines the treatment regimen, detailing the intervention’s duration, session frequency and length, and the overall hours of therapy. Summaries of the outcomes assessed in each study are also presented (Table 1).

### 3.2. Methodological Quality of the Included Studies

Analysis of the methodological quality in the 14 studies revealed the following. Randomization process: low risk of bias in 92.9% (13/14) and some risk in 7.1% (1/14). Intervention adherence: an even split with 50% (7/14) low risk and 50% (7/14) some risk. Missing outcome data: predominantly low risk at 78.6% (11/14), with some risk in 21.4% (3/14). Outcome measurement: mirroring randomization, 92.9% (13/14) low risk and 7.1% (1/14) some risk. Selective reporting: similar trends with 78.6% (11/14) low risk and 21.4% (3/14) some risk. Overall risk of bias: more significant concerns with 42.9% (6/14) low risk and 57.1% (8/14) some risk, as detailed in Figure S1. Although the randomization process and outcome measurement mostly indicated a lower risk of bias, intervention adherence and overall bias were problems that surfaced in more than half the studies. For comprehensive risk evaluations, see Table S4.

### 3.3. Primary Outcome: Rhythmic Music Therapy and Active Music Therapy with Singing Most Effective

**Figure 3:**
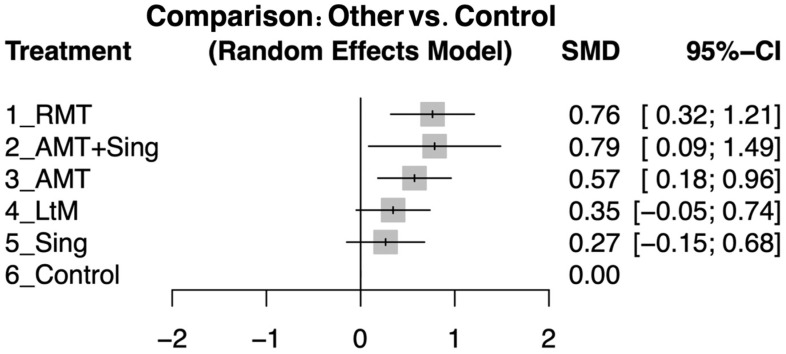
Forest plots showing the SMD in cognitive function improvement among various music therapy interventions compared to control groups in patients with dementia, following the intervention period.

This NMA assessed the impact of music therapy interventions on cognitive function in dementia patients. RMT showed a significant improvement (effect size: 0.76; 95% CI: 0.32 to 1.21), suggesting a robust, positive effect compared to the control group. The combined Active Music Therapy with Singing (AMT + Sing) also revealed a notable effect (effect size: 0.79; 95% CI: 0.09 to 1.49), though the wide confidence interval indicates some uncertainty in this estimate. AMT also yielded a moderate yet significant enhancement (effect size: 0.57; 95% CI: 0.18 to 0.96). The LtM intervention had a smaller, positive impact (effect size: 0.35; 95% CI: −0.05 to 0.74) but had a zero-crossed CI, indicating possible inconsistency. Simple singing activities (Sing) showed the smallest effect (effect size: 0.27; 95% CI: −0.15 to 0.68) and a zero-crossed CI that suggests uncertainty in its effectiveness. The control group served as a baseline for comparisons, with the effectiveness of interventions measured accordingly (Figure 3). Detailed pairwise comparisons between study arms, as detailed in individual studies, are depicted in Figure S2.

Table 2 presents the results from the pairwise meta-analyses above the diagonal line and the results from NMA below it. The effect size, represented by SMD, includes 95% CIs.

### 3.4. Secondary Outcome: Comparable Dropout Rates across Studies

**Figure 4:**
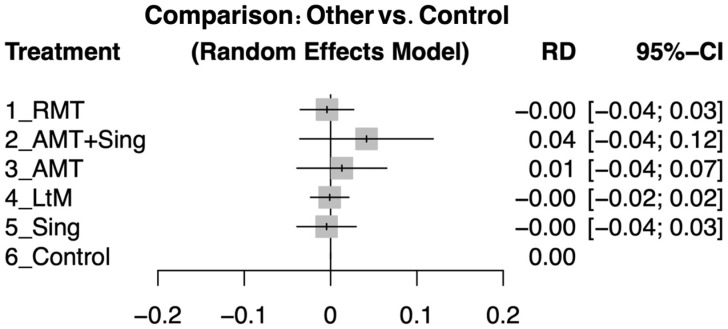
The forest plots show the risk difference (RD) in improving cognitive function in dementia patients among different music therapy interventions compared to control groups following the intervention period.

The findings indicated no significant differences in post-intervention dropout rates between various types of music therapy and the control group, with confidence intervals crossing zero for all groups (Figure 4). Detailed examinations of direct comparisons among study arms are outlined in specific studies (Figure S3).

### 3.5. Inconsistency Test

This network was constructed by establishing nodes and conducting both direct and indirect comparisons to assess consistency. The outcomes of inconsistency tests on the impact of various music therapy interventions on cognitive enhancement in dementia patients are available in Table S5. Information on dropout rates is provided in Table S6. Both sets of data reported *p*-values greater than 0.05, indicating no significant inconsistencies between the comparisons.

### 3.6. Sensitivity Analyses

In the sensitivity analysis excluding individual studies, data underscored the statistical significance of RMT, AMT + Sing, and AMT in enhancing cognitive function in dementia patients. The assessment of ranking and clinical impact across different music therapy interventions revealed a consistent trend, with RMT and AMT consistently offering significant benefits. For more detailed insights, refer to Figure S4 A–N.

During our alternative sensitivity evaluation, changing the pre–post correlation coefficient from 0.8 to 0.5 led to an updated network comparison (Figure S5). This adjustment confirmed that the direction of effect sizes, rankings of interventions, and overall interpretation of results were consistent with those obtained using the original 0.8 coefficients (Figure 3). These combined analyses underscore the reliability of our study’s findings, showcasing their stability in the face of selective study inclusion or exclusion and changes in assumed analytical values.

### 3.7. Publication Bias

The analysis of the funnel plot through Egger’s test resulted in a *p*-value of 0.144, suggesting there is no significant publication bias (Figure S6).

## 4. Discussion

### 4.1. Principal Results and Clinical Implications

Our NMA demonstrated that RMT and AMT + Sing were the most effective interventions for cognitive improvement in dementia patients. AMT alone was effective, while LtM and singing interventions alone had more negligible yet positive effects on cognitive function. Concerning dropout rates, there were no significant risk differences observed between various music interventions and the control group. This analysis offers essential insights for dementia patients and their caregivers, guiding therapeutic engagement. These results bolster the advocacy for music-based interventions, indicating that regular participation in such programs may yield meaningful improvements in cognitive function.

### 4.2. Importance of the Results in the Context of Current Research

Before our study, comprehensive meta-analyses had been published, such as the one by Dorris et al., 2021, in the *Journal of the American Geriatrics Society* [57]. This analysis compiled 21 studies conducted between 2010 and 2021 for a systematic review, with only 9 being meta-analyzed, involving 495 participants. Their findings indicated a small effect size (SMD = 0.30) of AMT on cognitive functions in elderly individuals with MCI or dementia. Another meta-analysis by Bian et al., 2021, published in *NeuroRehabilitation* [58], gathered seven studies prior to 2020 with a total of 455 participants. Additionally, Moreno-Morales et al., 2020, in a meta-analysis published in *Frontiers in Medicine* [59], included eight studies from 2010 to 2020, totaling 816 participants. Their study determined that music interventions have a beneficial impact on enhancing cognitive function. However, the authors also noted that due to the small sample sizes and insufficient evidence regarding types of music, the conclusions drawn from their analyses remain limited.

In our study, we concluded that RMT and AMT + Sing were the most effective types of music for cognitive training in dementia treatment, followed by AMT alone. This research is the first piece of literature to explore the effectiveness, comparison, and ranking of different types of music in the study of cognitive function in dementia. In our study, we directly compared and ranked the impacts of various music therapies on cognitive function in dementia, considering each music therapy as a benchmark for the study. Nonetheless, several studies are based on self-reported surveys and lack prospective designs that clearly define the types of music interventions used (Table S3), and while some systematic reviews have included patients with dementia during and after treatment, they fail to specify the exact types of music interventions [22,23,24,26,27,28]. In other words, our goal was not to answer whether all music interventions are effective for cognitive function in dementia patients but to evaluate the different impacts on the cognitive function of dementia patients undergoing different types of music, with these music types being part of the ranking results.

### 4.3. Possible Interpretations of Observations

In our investigation into the efficacy ranking of different types of music in improving dementia, we hypothesized that interactive musical activities might play a significant role. This is facilitated by the fundamental characteristic of neuroplasticity, which allows the brain to reorganize and repair itself [60].

Our review of studies on music programs for dementia patients suggests that the efficacy of RMT is likely linked to its emphasis on engaging in rhythm-based activities. The incorporation of rhythmic cues in music is instrumental in promoting movement, a key component in many neurorehabilitation approaches [61]. Furthermore, within the context of neurodegenerative disorders such as dementia, the melody, pitch, and harmony of music may offer therapeutic benefits through sensory–motor stimulation [62,63]. Secondly, AMT and Singing have demonstrated positive outcomes, with AMT engaging participants actively based on the principles of neuroplasticity. This engagement can bolster neuroplasticity through sensory involvement, emotional expression, and cognitive stimulation. Singing, on the other hand, can evoke memories and emotional responses, potentially activating neural networks associated with memory [48,50,51,52,53], for instance, using music as a stimulus to direct cognitive attention and sensory responses. The rhythmic and melodic elements of music may activate the brainstem reticular system and attention networks, helping patients with dementia maintain focus on external stimuli, thereby aiding in sensory integration and orientation [64]. Furthermore, attention control training in dementia is crucial, with studies highlighting the use of musical exercises to improve sustained, selective, and divided attention. By engaging auditory processing pathways and attention networks, music can strengthen the frontal systems involved in attention control, which are vital for cognitive function and often impaired in dementia [65,66]. Research also indicates that enhancing auditory perception training can improve auditory recognition and perception in patients with dementia. Complex auditory signals in music can stimulate the auditory cortex and related neural pathways, potentially leading to improved auditory processing and recognition, crucial for cognitive clarity and function [67,68]. Moreover, memory training through music utilizes the mnemonic potential of melody and rhythm. Familiar music can serve as a template for memory encoding and retrieval, leveraging preserved neural circuits related to long-term memory and emotional significance, which may remain intact during the progression of dementia [69,70]. Lastly, music has been noted to address psychosocial issues in dementia. Collective music-making and engagement can foster social interaction, reduce anxiety and depression, and improve the quality of life [71,72]. Enhancing emotional regulation and social connection indirectly supports cognitive health and resilience. To conclude, music therapy, particularly RMT and AMT, offers a viable non-pharmacological approach to mitigating cognitive deficits in patients with dementia. This effect is likely due to music’s engagement with the brain’s intrinsic capabilities governing movement, emotion, and cognition, thus providing a comprehensive intervention for multifaceted conditions like dementia.

### 4.4. Limitations

Our NMA has uncovered the potential benefits of music therapy in enhancing cognitive conditions in patients with dementia. However, some limitations must be acknowledged in interpreting our findings. The inclusion of patients from diverse populations and across various age groups may have introduced variability in the characteristics of dementia, complicating the analysis. Additionally, the use of the MMSE in some instances, especially for MCI, has lower sensitivity, necessitating further evaluation to provide a more comprehensive assessment of cognitive function [73,74]. Another notable issue is the increased dropout rates among the elderly, which may bias the outcomes. To confirm the trustworthiness of our study results, we meticulously reviewed the 14 studies included in our analysis. Through consistency checks and sensitivity analyses, we verified that no specific study or group of studies skewed the overall results. Despite these hurdles, our findings have significant implications for the everyday care and mental health of patients with dementia. Future research should aim to create standardized treatment protocols and perform long-term follow-up studies to thoroughly assess the impact of music interventions on cognitive functions in dementia.

## 5. Conclusions

In summary, our findings demonstrate that music therapy interventions such as RMT, AMT + Sing, and AMT not only significantly enhance cognitive functions in dementia patients but also maintain dropout rates at levels comparable to usual care. This underscores the efficacy and practicality of these interventions, confirming their value as viable options for dementia care.

## Figures and Tables

**Table 1:** Summary of the effectiveness of music therapy in improving cognitive function in dementia, including details of the conducted trials.

								Intervention Group		Control Group				Frequency of Treatment			
Authors and Year	Country	Study Design	Comparison	*n*	Dropouts	Age, Mean (SD)	Dementia Severity	Session Style	Type of Music	Control Type	Control Descriptions	Outcomes	Duration of Intervention	Period (Weeks)	Frequency (Times/Week)	Duration (Hours)	Total Hours
Biasutti et al., 2018 [53]	Italy	RCT	Music Control	18 17	3/21 3/20	83.39 (7.81) 83.76 (6.16)	Mild	AMT	Improvisation	Active	Soft gym	MMSE	70 min/once biweekly/12 weeks	≥12	<2	<24	7
Biasutti et al., 2021 [50]	Italy	RCT	Music Control	20 25	5/25 1/26	83.95 (7.84) 85.12 (6.14)	Mild	AMT	Improvisation	Active	Gymnastic activities	MMSE	70 min/twice a week/6 weeks	<12	≥2	<24	14
Ceccato et al., 2012 [51]	Italy	RCT	Music Control	27 23	0/27 0/23	85.50 (5.90) 87.20 (7.10)	Moderate	AMT	Special compositions	Waitlist	Standard care and Waitlist	MMSE	45 min/twice a week/24 weeks	≥12	≥2	≥24	36
Cheung et al., 2018 [44]	Hong Kong	Multi-RCT	MM LtM Control	45 40 39	13/58 14/54 14/53	85.71 (6.68) 84.50 (6.82) 85.58 (7.46)	Moderate	RMT LtM	Multiple music	Active	Social activity	MMSE	40 min/twice a week/6 weeks	<12	≥2	<24	8
Chu et al., 2014 [52]	Taiwan	RCT	Music Control	49 51	3/52 1/52	82.00 (6.80)	Moderate	AMT + Sing	Improvisation	Passive	Usual nursing home care	MMSE	30 min/twice a week/6 weeks	<12	≥2	<24	6
Giovagnoli et al., 2017 [46]	Italy	Multi-RCT	Music Control	13 13	4/17 4/17	73.92 (7.74) 73.50 (5.96)	Moderate	AMT	Improvisation	Active	Cognitive training	MMSE	45 min/twice a week/12 weeks	≥12	≥2	<24	18
Giovagnoli et al., 2018 [48]	Italy	Multi-RCT	Music Control	23 22	0/23 0/22	74.30 (5.70) 72.00 (7.30)	Moderate	AMT	Improvisation	Passive	Standard care	MMSE	45 min/twice a week/24 weeks	≥12	≥2	≥24	36
Lyu et al., 2018 [54]	China	RCT	Music Control	97 95	3/100 4/99	68.90 (7.10) 69.90 (7.90)	Mild-Severe	AMT	Patients‘ Preferences	Passive	Reading and Routine medical treatment	MMSE	30–40 min/twice a day/12 weeks	≥12	≥2	≥24	280
Pérez-Ros et al., 2019 [49]	Spain	RCT	Music Control	47 72	0/47 0/72	80.06 (7.63) 80.80 (7.36)	Moderate	LtM	Patients‘ Preferences	Passive	Standard care	MMSE	60 min/5 times a week/8 weeks	<12	≥2	≥24	40
Pongan et al., 2017 [47]	France	RCT	Music Control	31 28	0/31 0/28	78.80 (7.43) 80.20 (5.71)	Mild	Sing	Patients‘ Preferences	Active	Painting	FAB	120 min/once a week/12 weeks	≥12	<2	≥24	24
Prinz et al., 2023 [43]	Germany	RCT	Music Control	43 26	5/38 5/21	80.50 (5.76) 83.71 (6.34)	Mild-Severe	RMT	Old songs/Classical	Passive	Standard care	MMSE	45–60 min/twice a week/12 weeks	≥12	≥2	≥24	105
Tang et al., 2018 [45]	China	RCT	Music Control	38 39	0/38 0/39	76.36(4.94) 75.38(4.94)	Moderate	AMT	Old songs	Passive	Standard care	MMSE	50 min/3 time a week/12 weeks	≥12	≥2	≥24	30
van de Winckel et al., 2004 [55]	Belgium	RCT	Music Control	15 9	0/15 1/10	81.33(4.24) 81.90(4.18)	Moderate-Severe	AMT	Old songs	Active	Daily one-to-one conversation with therapist	MMSE	30 min/once for day/12 weeks	≥12	<2	<24	6
Wang et al., 2018 [56]	China	RCT	Music Control	30 30	0/30 0/30	70.40 (7.50) 69.10 (7.20)	Mild	PMT	Old songs	Passive	Treatment as usual	MMSE	30–50 min/3 time a day/12 weeks	≥12	≥2	≥24	288

Abbreviations: RCT: Randomized Controlled Trial; N: Number; MT: Music Therapy; MM: Music Movement; RMT: Rhythmic Music Therapy; AMT: Active Music Therapy; LtM: Listening to Music; MMSE: Mini-Mental Status Examination; FAB: Frontal Assessment Battery.

**Table 2:** Comparison and ranking of different music interventions aimed at improving cognitive function in patients with dementia.

RMT	-	-	0.36 [−0.32; 1.04]	-	0.76 [0.30; 1.23]
−0.02 [−0.85; 0.81]	AMT + Sing	-	-	-	0.79 [0.09; 1.49]
0.19 [−0.40; 0.78]	0.21 [−0.59; 1.01]	AMT	-	-	0.57 [0.18; 0.96]
0.42 [−0.10; 0.94]	0.44 [−0.36; 1.24]	0.23 [−0.32; 0.78]	LtM	-	0.32 [−0.08; 0.73]
0.50 [−0.11; 1.10]	0.52 [−0.29; 1.33]	0.31 [−0.26; 0.87]	0.08 [−0.49; 0.65]	Sing	0.27 [−0.15; 0.68]
0.76 [0.32; 1.21]	0.79 [0.09; 1.49]	0.57 [0.18; 0.96]	0.35 [−0.05; 0.74]	0.27 [−0.15; 0.68]	Control

## Data Availability

The data are included in the article and the Supplementary Files.

## Supplementary Materials

The following supporting information can be downloaded at: https://www.mdpi.com/article/10.3390/jpm14050497/s1, Figure S1: Summary of the quality assessment for included studies; Figure S2: Individual study results (with studies excluded) grouped by treatment comparison; Figure S3: Individual study results (with studies excluded) grouped by treatment comparison; Figure S4: The forest plots display the results of the sensitivity analysis conducted using the one-study removal method, involving 14 studies (labeled A to N). The ranking and clinical significance remain unchanged, indicating that the conclusions of our study are not affected by the inclusion or exclusion of any single study; Figure S5: Forest plot displaying the improvement in cognitive function in dementia patients after receiving different types of music therapy interventions, presented as standardized mean differences (SMDs). The pre-post correlation coefficient used in the calculation of data was changed from 0.8 used in Figure 3 to 0.5 in this figure as a sensitivity analysis. The ranking and clinical interpretations remained unchanged compared to Figure 3. This suggests that the conclusions of our study remain unchanged despite different assumptions regarding the coefficient used for transformation; Figure S6: Publication bias; Table S1: PRISMA for network meta-analysis checklist; Table S2: Keywords and search results in different databases; Table S3: Studies excluded from the analysis along with the reasons for their exclusion; Table S4: Detailed quality assessment of included studies using; Table S5: Inconsistency test outcomes for the standardized mean difference in enhancing cognitive function in patients with dementia treated of music therapy; Table S6: Inconsistency test results for the risk difference in dropout rates when applying music therapy to alleviate cognitive function in patients with dementia.
