# Supplementary material for: The Sound of Memory: Investigating Music Therapy’s Cognitive Benefits in Patients with Dementia—A Network Meta-Analysis of Randomized Controlled Trials
Source: J Pers Med. 2024 May 8;14(5):497. doi: 10.3390/jpm14050497 (PMC11122286; doi:10.3390/jpm14050497)
Supplement: Supplementary file 1 [file jpm-14-00497-s001.zip › jpm-2985970-supplementary.pdf]

Table S1 - PRISMA for network meta-analysis checklist

Section and Topic	#	Checklist item	Location
**Title**			
Title	1	Identify the report as a systematic review incorporating a network meta-analysis (or related form of meta-analysis).	Title
**Abstract**			
Structured summary	2	Provide a structured summary including, as applicable: Background: main objectives / Methods: data sources; study eligibility criteria, participants, and interventions; study appraisal; and synthesis methods, such as network meta-analysis. / Results: number of studies and participants identified; summary estimates with corresponding confidence/credible intervals; treatment rankings may also be discussed. Authors may choose to summarize pairwise comparisons against a chosen treatment included in their analyses for brevity. / Discussion/conclusions: limitations; conclusions and implications of findings. / Other: primary source of funding; systematic review registration number with registry name.	Abstract
**Introduction**			
Rationale	3	Describe the rationale for the review in the context of what is already known, including mention of why a network meta-analysis has been conducted.	Introduction / 1st and 2nd paragraph
Objectives	4	Provide an explicit statement of questions being addressed, with reference to participants, interventions, comparisons, outcomes, and study design (PICOS).	Introduction / 3rd paragraph
**Methods**			
Protocol & registration	5	Indicate whether a review protocol exists and where it can be accessed; and, if available, provide registration information, including registration number.	Materials and Methods/ 1st paragrph
Eligibility criteria	6	Specify study characteristics (e.g., PICOS, length of follow-up) and report characteristics (e.g., years considered, language, publication status) used as criteria for eligibility, giving	Materials and Methods/ Inculusion and exculusion

		rationale. Clearly describe eligible treatments included in the treatment network, and note whether any have been clustered or merged into the same node (with justification).	
Information sources	7	Describe all information sources (e.g., databases with dates of coverage, contact with study authors) in the search and date last searched.	Table S2
Search	8	Present full electronic search strategy for at least one database, including any limits used, such that it could be repeated.	Table S2
Study selection	9	State the process for selecting studies (i.e., screening, eligibility, included in systematic review, and, if applicable, included in the meta-analysis).	Materials and Methods / Study Identification / Inculusion and exculusion criteria
Data collection	10	Describe method of data extraction from reports (e.g., piloted forms, independently, in duplicate) and any processes for obtaining and confirming data.	Materials and Methods / Data extraction
Data items	11	List and define all variables for which data were sought (e.g., PICOS, funding sources) and any assumptions and simplifications made.	Materials and Methods / Data extraction and conversion
Network geometry	S1	Describe methods used to explore the geometry of the treatment network under study and potential biases related to it. This should include how the evidence base has been graphically summarized for presentation, and what characteristics were compiled and used to describe the evidence base to readers.	Materials and Methods / Modeling for network meta-analysis
Risk of bias within	12	Describe methods used for assessing risk of bias of individual studies (including specification of whether this was done at the study or outcome level), and how this information is to be used in any data synthesis.	Materials and Methods / Quality appraisal
Summary measures	13	State the principal summary measures (e.g., risk ratio, difference in means). Also describe the use of additional summary measures assessed, such as treatment rankings, as well as modified approaches used to present summary findings from meta-analyses.	Materials and Methods / Outcome
Planned methods of analysis	14	Describe the methods of handling data and combining results of studies for each network meta-analysis. This should include, but not be limited to: Handling of multi-arm trials;	Materials and Methods / Statistical analyses

		Selection of variance structure; Selection of prior distributions in Bayesian analyses; and Assessment of model fit.	
Assessment of inconsistency	S2	Describe the statistical methods used to evaluate the agreement of direct and indirect evidence in the treatment network(s) studied. Describe efforts taken to address its presence when found.	Materials and Methods / Statistical analyses
Risk of bias across	15	Specify any assessment of risk of bias that may affect the cumulative evidence.	Materials and Methods / Publication bias
Additional analyses	16	Describe methods of additional analyses if done, indicating which were pre-specified. This may include, but not be limited to, the following: Sensitivity or subgroup analyses; Meta-regression analyses; Alternative formulations of the treatment network; and Use of alternative prior distributions for Bayesian analyses (if applicable).	Materials and Methods / Sensitivity analyses
**Results**			
Study selection	17	Give numbers of studies screened, assessed for eligibility, and included in the review, with reasons for exclusions at each stage, ideally with a flow diagram.	Results / Study identification Figure 1, Table S2, Table S3
Network structure	S3	Provide a network graph of the included studies to enable visualization of the geometry of the treatment network.	Fugre 2
Network geometry	S4	Provide a brief overview of characteristics of the treatment network. This may include commentary on the abundance of trials and randomized patients for the different interventions and pairwise comparisons in the network, gaps of evidence in the treatment network, and potential biases reflected by the network structure.	Results / Network model formation / Figure 2
Study characteristics	18	For each study, present characteristics for which data were extracted (e.g., study size, PICOS, follow-up period) and provide the citations.	Table 1
Risk of bias within	19	Present data on risk of bias of each study and, if available, any outcome level assessment.	Table S4, Figure S1, Methodological quality

Results of individual studies	20	For all outcomes considered (benefits or harms), present, for each study: (1) simple summary data for each intervention group, and (2) effect estimates and confidence intervals. Modified approaches may be needed to deal with information from larger networks.	Table 1
Synthesis of results	21	Present results of each meta-analysis done, including confidence/credible intervals. In larger networks, authors may focus on comparisons versus a particular comparator (e.g. placebo or standard care), with full findings presented in an appendix. League tables and forest plots may be considered to summarize pairwise comparisons. If additional summary measures were explored (such as treatment rankings), these should also be presented.	Outcomes / Figure 3, Figure 4, Figure S2, Figure S3, Table 2
Exploration for inconsistency	S5	Describe results from investigations of inconsistency. This may include such information as measures of model fit to compare consistency and inconsistency models, P values from statistical tests, or summary of inconsistency estimates from different parts of the treatment network.	Inconsistency test<br>Table S5, Table S6
Risk of bias across	22	Present results of any assessment of risk of bias across studies for the evidence base being studied.	Publicaton bias, Figure S6
Additional analyses	23	Give results of additional analyses, if done (e.g., sensitivity or subgroup analyses, meta-regression analyses, alternative network geometries studied, alternative choice of prior distributions for Bayesian analyses, and so forth).	Sensitivity analysis / Figure S4, Figre S5
**Discussion**			
Summary of evidence	24	Summarize the main findings, including the strength of evidence for each main outcome; consider their relevance to key groups.	Discussion<br>Findings and implications
Limitations	25	Discuss limitations at study and outcome level (e.g., risk of bias), and at review level (e.g., incomplete retrieval of identified research, reporting bias). Comment on the validity of the assumptions, such as transitivity and consistency. Comment on any concerns regarding network geometry (e.g., avoidance of certain comparisons).	Discussion<br>Linitations

Conclusions	26	Provide a general interpretation of the results in the context of other evidence, and implications for future research.	Conclusion
**Funding**			
Funding	27	Describe sources of funding for the systematic review and other support (e.g., supply of data); role of funders for the systematic review. This should also include information regarding whether funding has been received from manufacturers of treatments in the network and/or whether some of the authors are content experts with professional conflicts of interest that could affect use of treatments in the network.	Funding

Table S2 - Keywords and search results in different databases **1380**

Database	Keyword	Date	Results
PubMed	('music') AND ('cognitive') AND ('dementia' OR 'alzheimer') AND ('random' OR 'randomized' OR 'randomised')	2024.01.15	163
Embase	('music') AND ('cognitive') AND ('dementia' OR 'alzheimer') AND ('random' OR 'randomized' OR 'randomised')	2024.01.15	238
Cochrane CENTRAL	('music') AND ('cognitive') AND ('dementia' OR 'alzheimer') AND ('random' OR 'randomized' OR 'randomised')	2024.01.15	203
Web of Science	('music') AND ('cognitive') AND ('dementia' OR 'alzheimer') AND ('random' OR 'randomized' OR 'randomised')	2024.01.15	173
			777

Database	Keyword	Date	Results
PubMed	('sing') AND ('cognitive') AND ('dementia' OR 'alzheimer') AND ('random' OR 'randomized' OR 'randomised')	2024.01.15	24
Embase	('sing') AND ('cognitive') AND ('dementia' OR 'alzheimer') AND ('random' OR 'randomized' OR 'randomised')	2024.01.15	5
Cochrane CENTRAL	('sing') AND ('cognitive') AND ('dementia' OR 'alzheimer') AND ('random' OR 'randomized' OR 'randomised')	2024.01.15	40
Web of Science	('sing') AND ('cognitive') AND ('dementia' OR 'alzheimer') AND ('random' OR 'randomized' OR 'randomised')	2024.01.15	23
			92

Database	Keyword	Date	Results

PubMed	('rhythm') AND ('cognitive') AND ('dementia' OR 'alzheimer') AND ('random' OR 'randomized' OR 'randomised')	2024.01.15	97
Embase	('rhythm') AND ('cognitive') AND ('dementia' OR 'alzheimer') AND ('random' OR 'randomized' OR 'randomised')	2024.01.15	203
Cochrane CENTRAL	('rhythm') AND ('cognitive') AND ('dementia' OR 'alzheimer') AND ('random' OR 'randomized' OR 'randomised')	2024.01.15	115
Web of Science	('rhythm') AND ('cognitive') AND ('dementia' OR 'alzheimer') AND ('random' OR 'randomized' OR 'randomised')	2024.01.15	96
			511

Table S3: Studies excluded from the analysis along with the reasons for their exclusion.

No.	First Author / Year	Title	Journal/Book	Exclusion reasons
1	Bakerjian et al., 2020	The Impact of Music and Memory on Resident Level Outcomes in California Nursing Homes	Journal of the American Medical Directors Association	Not RCT
2	Buard et al., 2021	Randomized controlled trial of neurologic music therapy in Parkinson's disease: research rehabilitation protocols for mechanistic and clinical investigations	Trials	Protocol
3	Cheung et al., 2018	The effects of the music-with-movement intervention on the cognitive functions of people with moderate dementia: a randomized controlled trial	Aging Ment Health	Incomplete data
4	Cheung et al., 2022	A home-based dyadic music-with-movement intervention for people with dementia and caregivers: A hybrid type 2 cluster-randomized effectiveness-implementation design	Clinical Interventions in Aging	No relevant outcome data
5	Clark et al., 2020	"It's Feasible to Write a Song": A Feasibility Study Examining Group Therapeutic Songwriting for People Living With Dementia and Their Family Caregivers	Frontiers in Psychology	Incomplete data
6	Cooke et al. 2010	A randomized controlled trial exploring the effect of music on agitated behaviours and anxiety in older people with dementia	Aging & Mental Health	Incomplete data
7	Davison et al., 2016	A personalized multimedia device to treat agitated behavior and improve mood in people with dementia: A pilot study	Geriatr Nursing	Pilot study
8	Delphin-Combe et al., 2013	Effect of a non-pharmacological intervention, Voix d'Or(®), on behavior disturbances in Alzheimer disease and associated disorders	Gériatrie et Psychologie Neuropsychiatrie du Vieillissement	No relevant outcome data
9	Dimitriou et al., 2020	Non-Pharmacological interventions for the anxiety in patients with dementia. A cross-over randomised controlled trial	Behavioural Brain Research	No relevant outcome data
10	Feng et al., 2020	Effects of choral singing versus health education on cognitive decline and aging: a randomized controlled trial	Aging-US: Peer-Reviewed Aging Research Journal	Incomplete data

11	Gaviola et al., 2020	Impact of individualised music listening intervention on persons with dementia: A systematic review of randomised controlled trials	Australasian journal on ageing	Review & meta analysis
12	Giovagnoli et al., 2017	Cognitive training in Alzheimer's disease: a controlled randomized study	Neurological Sciences	No relevant outcome data
13	Guétin et al., 2009	Effect of music therapy on anxiety and depression in patients with Alzheimer's type dementia: randomised, controlled study	Dementia and Geriatric Cognitive Disorders	No relevant outcome data
14	Jung et al., 2023	Effect of internet-based vs. in-person multimodal interventions on patients with mild to moderate Alzheimer's disease: a randomized, cross-over, open-label trial	Frontiers in Public Health	No music intervention
15	Kwak et al., 2020	Findings From a Prospective Randomized Controlled Trial of an Individualized Music Listening Program for Persons With Dementia	Journal of Applied Gerontology	Incomplete data
16	Lai et al., 2016	Interdisciplinary collaboration in the use of a music-with-movement intervention to promote the wellbeing of people with dementia and their families: Development of an evidence-based intervention protocol	Nursing & Health Sciences	Protocol
17	Liu et al., 2021	Group Music Intervention Using Percussion Instruments to Reduce Anxiety Among Elderly Male Veterans with Alzheimer Disease	Medical Science Monitor	No relevant outcome data
18	Loi et al., 2022	Music and Psychology & Social Connections Program: Protocol for a Novel Intervention for Dyads Affected by Younger-Onset Dementia	Brain Sciences	Protocol
19	Mahendran et al., 2017	Art therapy and music reminiscence activity in the prevention of cognitive decline: Study protocol for a randomized controlled trial	Trials	Protocol
20	McCreedy et al., 2022	Pragmatic Trial of Personalized Music for Agitation and Antipsychotic Use in Nursing Home Residents With Dementia	Journal of the American Medical Directors Association	Incomplete data
21	McCreedy et al., 2022	Is there a role for music therapy in the recovery approach in mental health?	The Arts in Psychotherapy	No relevant outcome data
22	Moreira et al., 2023	Music Therapy Enhances Episodic Memory in Alzheimer's and Mixed Dementia: A Double-Blind Randomized Controlled Trial	Healthcare	No relevant outcome data

23	Na et al., 2019	A systematic review and meta-analysis of nonpharmacological interventions for moderate to severe dementia	Psychiatry Investigation	Review & meta analysis
24	Noone et al., 2019	Meta-analysis of psychosocial interventions for people with dementia and anxiety or depression	Aging & mental health	Review & meta analysis
25	Pongan et al., 2017	Can Musical or Painting Interventions Improve Chronic Pain, Mood, Quality of Life, and Cognition in Patients with Mild Alzheimer's Disease? Evidence from a Randomized Controlled Trial	Journal of Alzheimer's Disease	No relevant outcome data
26	Raglio et al., 2008	Efficacy of music therapy in the treatment of behavioral and psychiatric symptoms of dementia	Alzheimer Disease & Associated Disorders	Incomplete data
27	Raglio et al., 2008	Efficacy of music therapy in the treatment of behavioral and psychiatric symptoms of dementia	Alzheimer Dis Assoc Disord	No relevant outcome data
28	Raglio et al., 2015	Effect of Active Music Therapy and Individualized Listening to Music on Dementia: A Multicenter Randomized Controlled Trial	Journal of the American Geriatrics Society	No relevant outcome data
29	Raglio et al., 2015	Effects of music and music therapy on mood in neurological patients	World Journal of Psychiatry	Review & meta analysis
30	Ridder et al., 2002	Individual music therapy for agitation in dementia: an exploratory randomized controlled trial	Aging & Mental Health	No relevant outcome data
31	Sánchez et al., 2016	Comparing the Effects of Multisensory Stimulation and Individualized Music Sessions on Elderly People with Severe Dementia: A Randomized Controlled Trial	Journal of Alzheimer's Disease	Incomplete data
32	Sánchez et al., 2016	Comparing the Effects of Multisensory Stimulation and Individualized Music Sessions on Elderly People with Severe Dementia: A Randomized Controlled Trial	Journal of Alzheimer's Disease	No relevant outcome data
33	Särkämö et al., 2014	Music perception and cognition: development, neural basis, and rehabilitative use of music	Wiley Interdisciplinary Reviews: Cognitive Science	Incomplete data

34	Särkämö et al., 2014	Cognitive, Emotional, and Social Benefits of Regular Musical Activities in Early Dementia: Randomized Controlled Study	The Gerontologist	Incomplete data
35	Scott S. et al., 2016	A scoping review of music and anxiety, depression and agitation in older people with dementia in residential facilities and specialist care units	European Geriatric Medicine	Review & meta analysis
36	Sung et al., 2010	A preferred music listening intervention to reduce anxiety in older adults with dementia in nursing homes	Journal of clinical nursing	No relevant outcome data
37	Sung et al., 2012	A group music intervention using percussion instruments with familiar music to reduce anxiety and agitation of institutionalized older adults with dementia	International Journal of Geriatric Psychiatry	No relevant outcome data
38	Svansdottir et al., 2006	Music therapy in moderate and severe dementia of Alzheimer's type: a case-control study	International Psychogeriatrics	Incomplete data
39	Tan et al., 2018	Study protocol for a randomized controlled trial of choral singing intervention to prevent cognitive decline in at-risk older adults living in the community	Frontiers in Aging Neuroscience	Protocol
40	Tang et al., 2013	The effectiveness of nursing management on improving health outcomes for hospitalized older adults with delirium: A systematic review protocol	JBI Database of Systematic Reviews and Implementation Reports	Protocol
41	Thornley et al., 2016	Music therapy in patients with dementia and behavioral disturbance on an inpatient psychiatry unit: results from a pilot randomized controlled study	International Psychogeriatrics	Pilot study
42	Valdiglesias et al., 2017	Is Salivary Chromogranin A a Valid Psychological Stress Biomarker During Sensory Stimulation in People with Advanced Dementia?	Journal of Alzheimer's Disease	Incomplete data
43	Vink et al., 2012	Effectiveness of group music intervention against agitated behavior in elderly persons with dementia	International Journal of Geriatric Psychiatry	No relevant outcome data
44	Zhang et al., 2023	Does music intervention relieve depression or anxiety in people living with dementia? A systematic review and meta-analysis	Aging & Mental Health	Review & meta analysis

Table S4 - Detailed quality assessment of included studies using
Cochrane risk of bias 2 tool

First author & Year	Randomization process	Intervention adherence	Missing outcome data	Outcome measurement	Selective reporting	Overall RoB
Biasutti et al., 2018	S	S3	S	S	L	S
Biasutti et al., 2021	L	S2	S	L	S	S
Ceccato et al., 2012	L	S3	L	L	S	S
Cheung et al., 2018	L	L2	L	L	S	L
Chu et al., 2013	L	L2	S	L	L	S
Giovagnoli et al., 2017	L	S2	S	L	L	L
Giovagnoli et al., 2018	L	S2	S	L	S	S
Lyu et al., 2018	L	S2	L	L	L	S
Pérez-Ros et al., 2019	S	S2	S	L	S	S
Pongan et al., 2017	L	S3	S	S	S	S
Prinz et al., 2023	S	S3	L	S	L	S
Tang et al., 2018	L	L2	L	L	L	L
van de Winckel et al., 2004	S	S2	S	S	S	S
Wang et al., 2018	L	S3	S	L	S	S

1 The study employed a waitlist control group design, which resulted in a more balanced comparison among different groups.
2 The differences in protocols among various groups may affect adherence and outcome.
3 Both groups were randomized to receive exercise interventions, and the study design utilized a balanced protocol, which minimized the impact on adherence.
H, high risk of bias; L, low risk of bias; S, some risk of bias.

Table S5. Inconsistency test outcomes for the standardized mean difference in enhancing cognitive function in patients with dementia treated of music therapy

Comparison	Studies	NMA	Direct	Indirect	Difference	95CI_L	95CI_U	p-Value
AMT:AMT+Sing	0.00	-0.21	-	-0.21	-	-	-	-
AMT:Control	5.00	0.57	0.57	-	-	-	-	-
AMT:LtM	0.00	0.23	-	0.23	-	-	-	-
AMT:RMT	0.00	-0.19	-	-0.19	-	-	-	-
AMT:Sing	0.00	0.31	-	0.31	-	-	-	-
AMT+Sing:Control	1.00	0.79	0.79	-	-	-	-	-
AMT+Sing:LtM	0.00	0.44	-	0.44	-	-	-	-
AMT+Sing:RMT	0.00	0.02	-	0.02	-	-	-	-
AMT+Sing:Sing	0.00	0.52	-	0.52	-	-	-	-
LtM:Control	3.00	0.35	0.32	0.73	-0.41	-2.17	1.35	0.65
RMT:Control	3.00	0.76	0.76	0.77	-0.01	-1.60	1.59	0.99
Sing:Control	3.00	0.27	0.27	-	-	-	-	-
LtM:RMT	1.00	-0.42	-0.36	-0.50	0.14	-0.91	1.19	0.79
LtM:Sing	0.00	0.08	-	0.08	-	-	-	-
RMT:Sing	0.00	0.50	-	0.50	-	-	-	-

95CI-L: lower limit of 95% confidence interval; 95CI-U: upper limit of 95% confidence interval; NMA: network meta-analysis.

Table S6. Inconsistency test results for the risk difference in dropout rates when applying music therapy to alleviate cognitive function in patients with dementia.

Comparison	Studies	NMA	Direct	Indirect	Difference	95CI-L	95CI-U	p-value
AMT:AMT+Sing	0	-0.03	-	-0.03	-	-	-	-
AMT:Control	5	0.01	0.01	-	-	-	-	-
AMT:LtM	0	0.01	-	0.01	-	-	-	-
AMT:RMT	0	0.02	-	0.02	-	-	-	-
AMT:Sing	0	0.02	-	0.02	-	-	-	-
AMT+Sing:Control	1	0.04	0.04	-	-	-	-	-
AMT+Sing:LtM	0	0.04	-	0.04	-	-	-	-
AMT+Sing:RMT	0	0.05	-	0.05	-	-	-	-
AMT+Sing:Sing	0	0.05	-	0.05	-	-	-	-
LtM:Control	3	0.00	0.00	-0.21	0.21	-0.10	0.53	0.19
RMT:Control	3	0.00	0.00	0.00	0.00	-0.09	0.08	0.91
Sing:Control	3	0.00	0.00	-	-	-	-	-
LtM:RMT	1	0.00	0.00	0.02	-0.02	-0.10	0.06	0.67
LtM:Sing	0	0.00	-	0.00	-	-	-	-
RMT:Sing	0	0.00	-	0.00	-	-	-	-

95CI-L: lower limit of 95% confidence interval; 95CI-U: upper limit of 95% confidence interval; NMA: network meta-analysis.

Figure S1: Summary of the quality assessment for included studies.

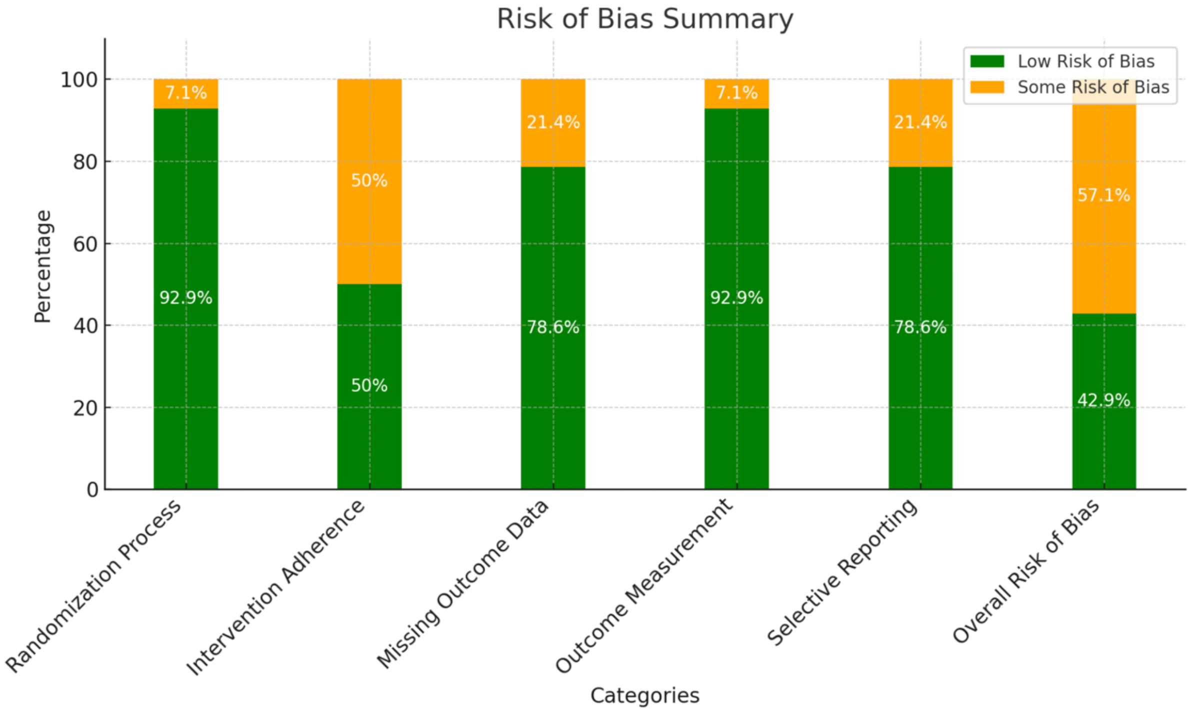

Figure S1 presents the risk of bias summary for the studies assessed in a meta-analysis. The categories evaluated include the randomization process, intervention adherence, missing outcome data, outcome measurement, selective reporting, and the overall risk of bias. The largest concerns appear in the categories of intervention adherence and overall risk of bias, where 50% of the studies exhibit some risk of bias. Notably, the outcome measurement category shows a high level of confidence, with 92.9% of studies classified as having a low risk of bias. This chart underscores the areas where the included studies have robust methodology and where potential biases might influence the validity of the findings.

Figure S2

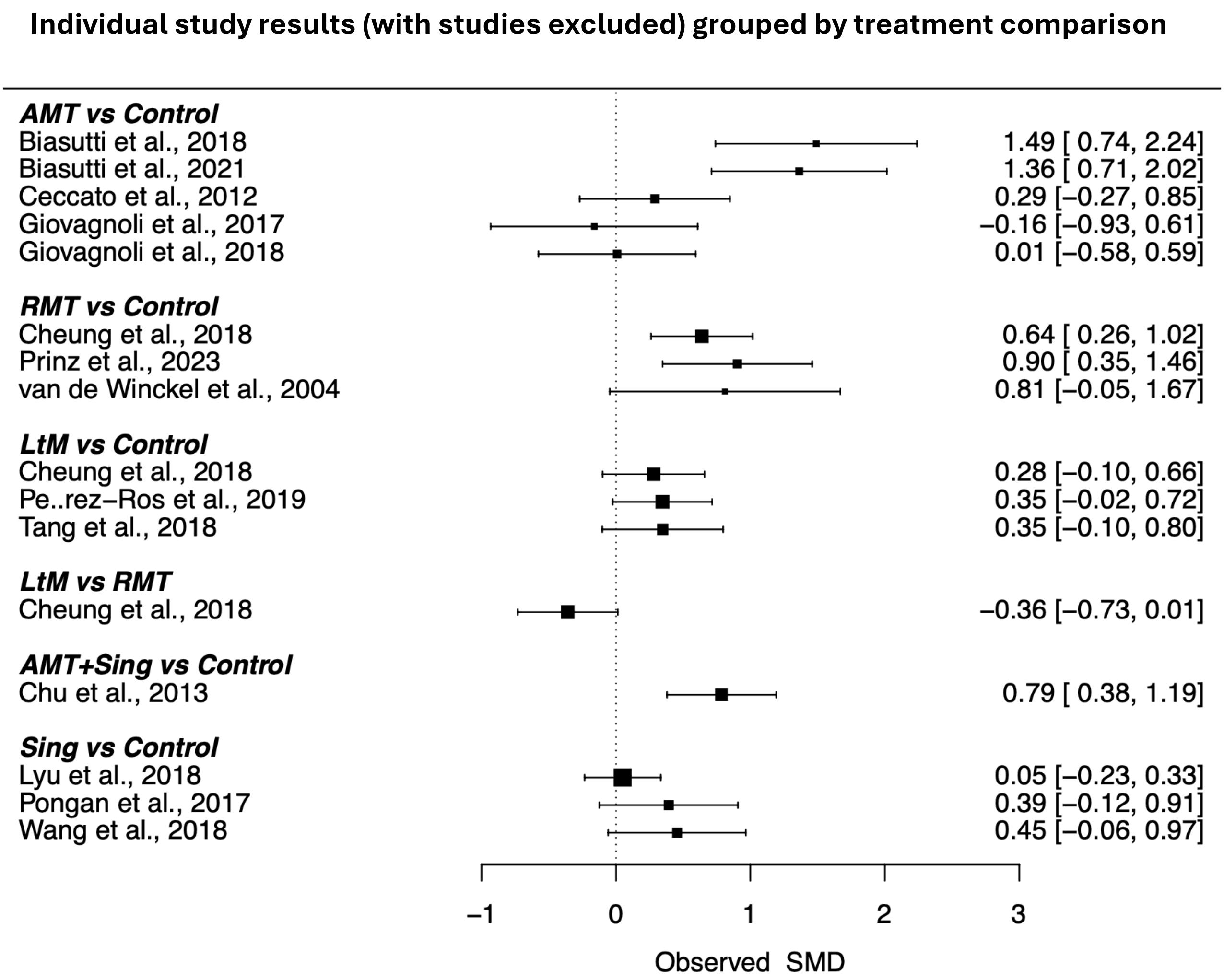

Fig. S2 - The forest plot of pairwise comparisons for different music therapy interventions to improve cognitive function in dementia patients, retrieved from the included trials, demonstrates the standardized mean difference (SMD).

Figure S3

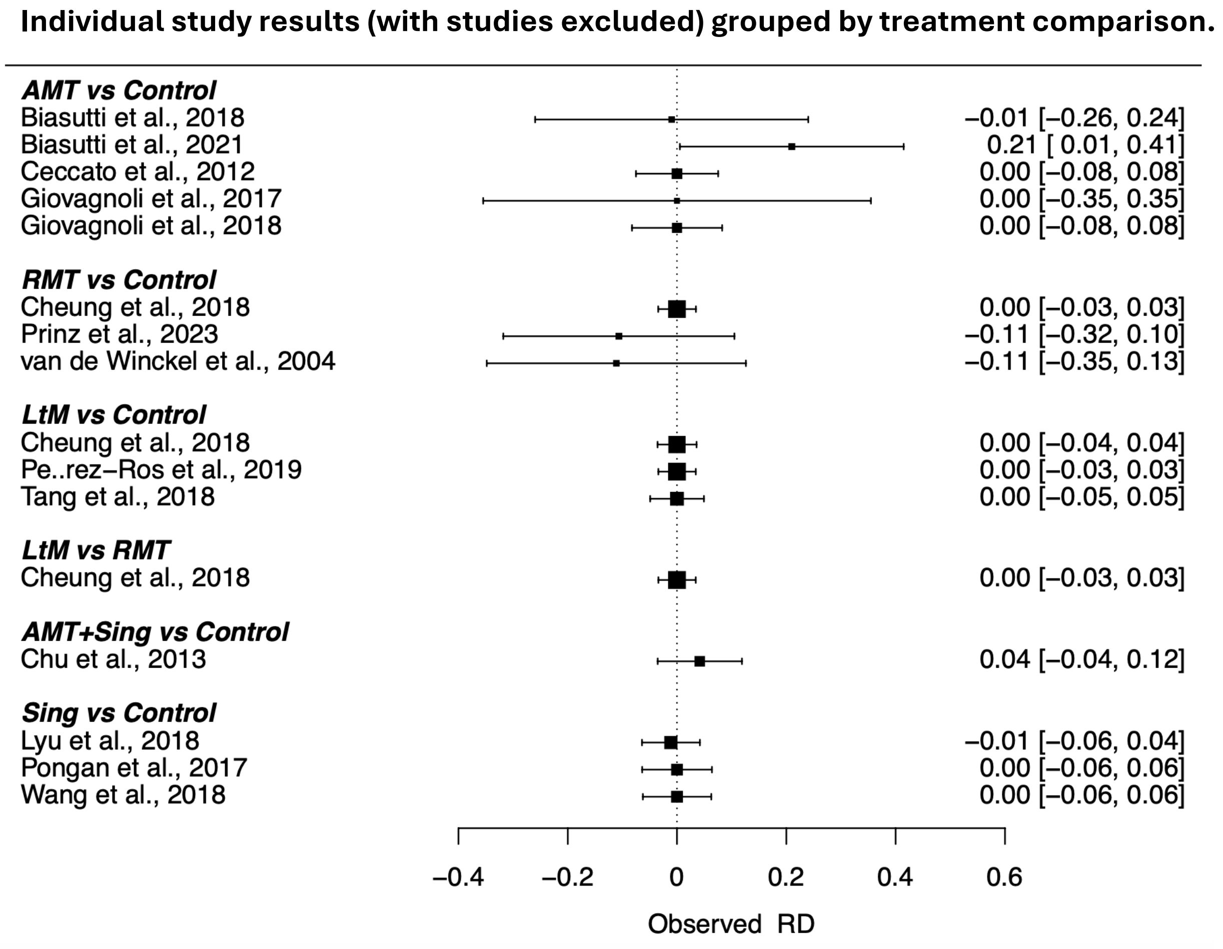

Fig. S3 - The forest plot of pairwise comparisons for different music therapy interventions to enhance cognitive function in dementia patients, retrieved from the included trials, demonstrates the risk difference (RD) of dropout rates. None of the comparisons reached statistical significance.

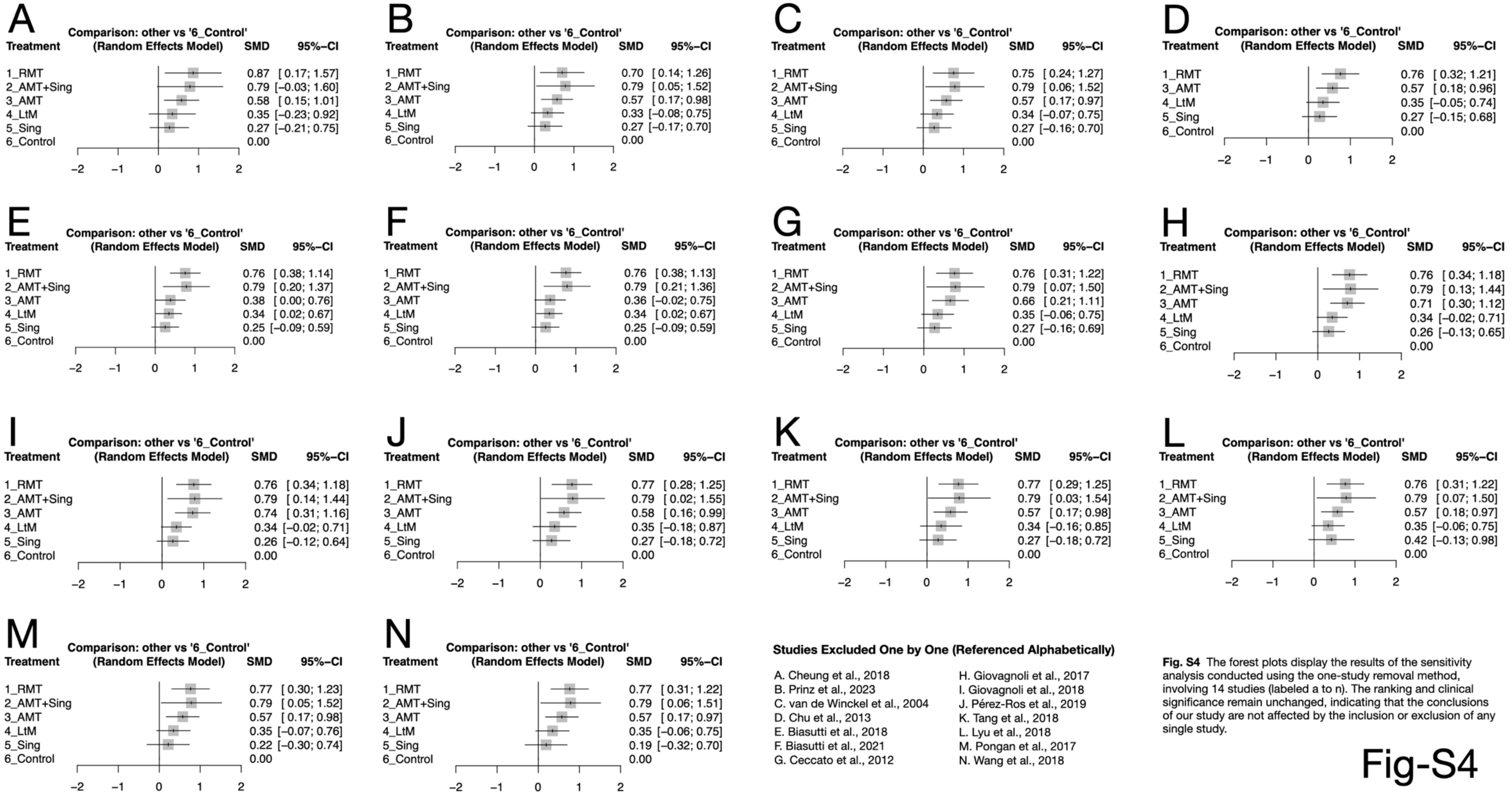

Fig. S4 - The forest plots display the results of the sensitivity analysis conducted using the one-study removal method, involving 14 studies (labeled A to N). The ranking and clinical significance remain unchanged, indicating that the conclusions of our study are not affected by the inclusion or exclusion of any single study.

Figure S5

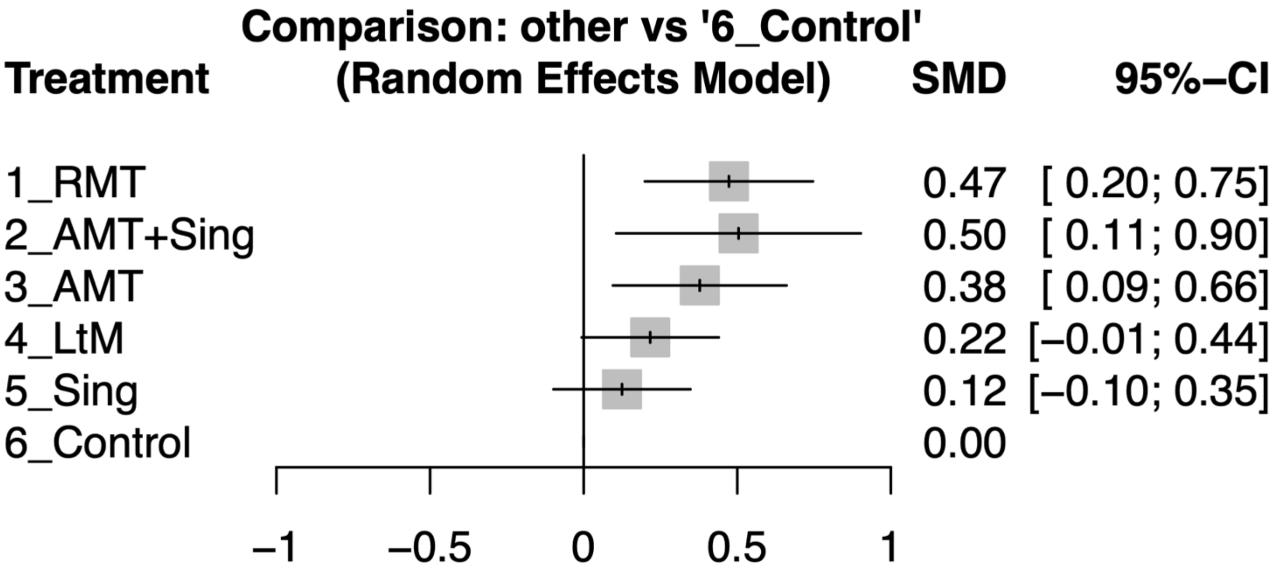

Fig. S5 - Forest plot displaying the improvement in cognitive function in dementia patients after receiving different types of music therapy interventions, presented as standardized mean differences (SMDs). The pre-post correlation coefficient used in the calculation of data was changed from 0.8 used in Figure 3 to 0.5 in this figure as a sensitivity analysis. The ranking and clinical interpretations remained unchanged compared to Figure 3. This suggests that the conclusions of our study remain unchanged despite different assumptions regarding the coefficient used for transformation.

Figure S6: Publication bias.

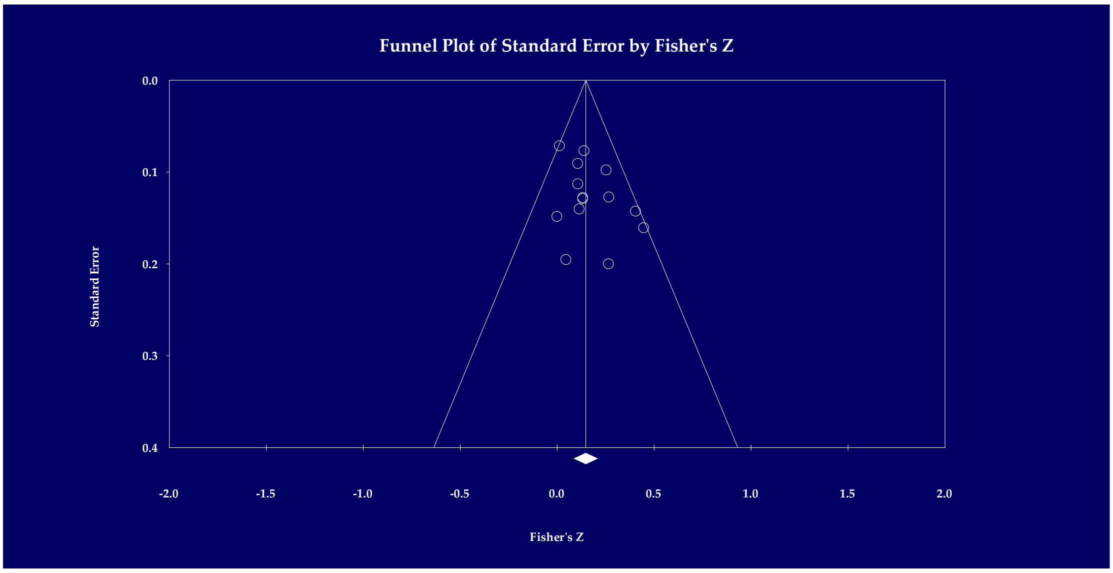

Egger's Test of the Intercept

In this case the intercept (B0) is 1.46, 95% CI (-0.47 to 3.38), with t=1.66, df=12. The 1-tailed p-value is 0.07, and the 2-tailed p-value is 0.13.
